# Editorial: Studying rare diseases using induced pluripotent stem cell (iPSC)-based model systems

**DOI:** 10.3389/fcell.2025.1686438

**Published:** 2025-09-08

**Authors:** Kevin R. Francis, Guokai Chen, Erkan Kiris

**Affiliations:** ^1^ Cellular Therapies and Stem Cell Biology Group, Sanford Research, Sioux Falls, SD, United States; ^2^ Department of Pediatrics, Sanford School of Medicine, University of South Dakota, Sioux Falls, SD, United States; ^3^ Faculty of Health Sciences, University of Macau, Taipa, Macao SAR, China; ^4^ Chinese Medicine and Translational Medicine R&D center, Zhuhai UM Science and Technology Research Institute, Zhuhai, Guangdong, China; ^5^ MoE Frontiers Science Center for Precision Oncology, University of Macau, Taipa, Macao SAR, China; ^6^ Department of Biological Sciences, Middle East Technical University, Ankara, Türkiye

**Keywords:** rare diseases, induced pluripotent stem cells, disease modelling, drug discovery, toxicity assays

## 1 Introduction

The diagnosis and study of rare diseases are complicated by their highly diverse nature, with an estimated total number of distinct rare diseases between 7,000-10,000 (Haendel et al., 2019; The Lancet Global, 2024). Moreover, less than 10% of these diseases have approved therapies (Amaral, 2022). Although each disease is classified individually as rare, their cumulative prevalence becomes substantial. It has been estimated that 263–446 million individuals worldwide suffer from a rare disease, while the global prevalence is calculated to be between 3.5% and 6% (Nguengang Wakap et al., 2020). Rare diseases exert a significant financial burden on healthcare systems globally, as the per-patient-per-year healthcare cost of rare diseases is significantly greater (up to 10x) than more common diseases (Tisdale et al., 2021; Andreu et al., 2023).

There is an unmet scientific need for both elucidation of the molecular mechanisms and development of therapeutics for rare diseases. To facilitate relevant scientific developments, it is critical that human cells, especially cell types affected in specific rare diseases, be obtained on a large scale for scientific work. To this end, human induced pluripotent stem cell (iPSC)-based platforms offer scalable, renewable, physiologically relevant, and patient-specific preclinical models (Rowe and Daley, 2019). A growing body of literature has demonstrated that human iPSCs provide valuable insights into the pathogenesis of various rare diseases and have facilitated the identification of potential therapeutic targets (Anderson and Francis, 2018). However, the majority of rare diseases remain poorly understood at the mechanistic level, which highlights the need for additional iPSC-based studies.

**FIGURE 1:**
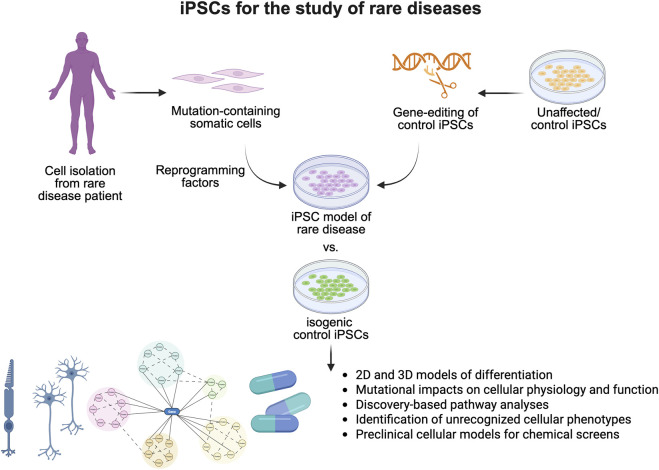
Induced pluripotent stem cells are essential models for the study of rare diseases. Generation of iPSC models of rare disease through reprogramming of patient cell types or generation of disease-causing mutations in unaffected iPSCs allows for the study of poorly characterized rare diseases within two-dimensional and three-dimensional models. Studies have demonstrated their utility in identifying novel cell physiology, signaling deficits, and cellular phenotypes associated with rare diseases, improving our understanding of disease pathogenesis, and serving as critical preclinical models for therapeutic development.

Approximately 80% of rare diseases have a genetic origin (The Lancet Global, 2024), which makes patient-derived iPSCs and isogenic controls unique model systems for their study (Figure 1). In addition to mechanistic studies, *in vitro* human disease models are also valuable for drug discovery and toxicity studies. Recent policy changes have also highlighted the utility of iPSC-based model systems for rare diseases. More specifically, the FDA Modernization Act 2.0, which allows therapeutics to be tested in cell-based assays without the need for animal testing for graduation to clinical trials, will likely increasingly drive interest in iPSC-based models for rare disease studies.

This Research Topic is aimed at compiling cutting-edge iPSC research and insightful reviews that significantly contribute to the advancement of iPSC-based rare disease research. The goal is to address the use of iPSCs or iPSC-derived cellular models in 2D or 3D formats for modeling rare diseases, drug discovery, and toxicity studies. This Research Topic includes three review articles and two original research articles that focus on distinct diseases, but all highlight the valuable utility of iPSC-based model systems for rare disease research.

## 2 Utility of human 2D or 3D models derived from iPSCs and isogenic controls for rare disease research

A unique contribution to this Research Topic is a research article focusing on Juvenile Nephronophthisis (NPH), a genetic disease affecting the kidney without effective treatments. Arai et al. developed the first human NPH disease models using patient-derived iPSCs, gene-edited iPSCs, and differentiated kidney organoids to better understand the underlying molecular mechanisms of the disease (Arai et al.). The authors demonstrated that NPHP1-deficient iPSCs exhibit abnormal cell proliferation, abnormalities in primary cilia, and renal cyst formation in iPSC-derived kidney organoids. Importantly, reintroduced NPHP1 expression reversed the cyst formation observed in organoids. This study demonstrated that iPSC-based model systems provide novel insights into the NPH disease mechanism, which can open doors for therapeutic development. Another great contribution to this Research Topic is a research article demonstrating the development and characterization of retinal organoids for the study of a rare form of autosomal dominant retinitis pigmentosa (RDH12-AD) (Méjécase et al.). Organoids from a patient carrying a dominant disease-causing variant in RDH12 exhibited normal RDH12 localization in early stages of development (up to week 44). By week 37, however, RDH12 retinal organoids exhibited both a reduction in photoreceptor number and shortened photoreceptor length. Furthermore, disruptions in cone function, retinol biosynthesis, and the vitamin A pathway were also evident in RDH12 models at week 44. This is the first published human disease model for RDH12-AD, reflecting the late-onset, milder disease course seen in patients. This study offers valuable insight into disease mechanisms and potential therapeutic targets through the use of iPSC-based model systems.

Three review articles in this Research Topic highlight the importance of iPSC-based models for selected rare diseases. Leith et al. provide a comprehensive overview of Usher syndrome, an inherited disorder primarily characterized by hearing loss and vision issues, and discuss recent advancements in the development of new preclinical models, including patient-specific iPSCs and organoid models (Leith et al.). A second review by Aalders et al. offers an in-depth review of the utility of human iPSCs for modeling Marfan syndrome, a multi-system disorder caused by mutations in the FBN1 gene (Aalders et al.). This review article highlights recent work demonstrating that iPSC-based models can successfully replicate disease features *in vitro*, offering valuable insights into disease mechanisms and the potential for discovering new therapies. Finally, Parvatam et al. discussed complex *in vitro* models (CIVM), including iPSCs, organoids, and organs-on-chip models, as powerful tools for developing effective therapies with a higher clinical translation chance (Parvatam et al.). The review discusses how CIVMs can improve the development of affordable and efficient rare disease treatments, emphasizing the use of policy and regulation to promote iPSC models and drug development for rare disease research.

## 3 Perspectives

In summary, this Research Topic generated two original research articles and three comprehensive reviews on the utility of human 2D or 3D models derived from iPSCs and isogenic controls for rare disease research. It is well appreciated that iPSC-based human model systems offer unique insights into rare disease research (Anderson and Francis, 2018). Ongoing use of patient-derived iPSCs can help clarify disease-associated mechanisms and facilitate the development of novel therapeutic options, a critical endeavor given that approximately 94% of rare diseases currently lack approved treatments (Amaral, 2022) (Figure 1). However, it is also important to recognize that the great potential of iPSC-based model systems demands further technological advances beyond common lineage-specific differentiation and 3D-organoid models. Recent studies in single-cell sequencing demonstrated more molecular signatures of specific cell types as well as the complex intercellular communications among different cells *in vivo*. Developing methods for the precise induction of cellular complexes and functional tissues would enable more accurate disease modeling. Meanwhile, the symptoms of various rare diseases emerge at different stages of life, highlighting the importance of the maturity of specific cell types. Developing novel technologies to precisely control the maturation of specific cell types would, therefore, be highly beneficial for both drug screening and mechanistic studies. Lastly, although not covered in this Research Topic, iPSC-based cell replacement therapies could be a viable option for the treatment of specific cellular phenotypes in rare diseases, considering the potential of iPSC-based cell therapies in regenerative medicine (Takahashi, 2025). Overall, rare diseases present a significant health challenge worldwide, and addressing them effectively requires strong international collaboration (Taruscio and Gahl, 2024). iPSC-based approaches for rare diseases should receive growing support from public and private institutions and patient advocacy groups globally.
